# Paracetamol (Acetaminophen) and its Effect on the Developing Mouse Brain

**DOI:** 10.3389/ftox.2022.867748

**Published:** 2022-03-22

**Authors:** Gaëtan Philippot, Kimia Hosseini, Armine Yakub, Yasser Mhajar, Mariam Hamid, Sonja Buratovic, Robert Fredriksson

**Affiliations:** ^1^ Department of Pharmaceutical Biosciences, Molecular Neuropharmacology, Biomedical Center, Uppsala University, Uppsala, Sweden; ^2^ Department of Organismal Biology, Environmental Toxicology, Evolutionary Biology Centre, Uppsala University, Uppsala, Sweden

**Keywords:** analgeic, paracetamol, developmental toxicity, neurotoxcity, mice

## Abstract

Paracetamol, or acetaminophen (AAP), is the most commonly used analgesic during pregnancy and early life. While therapeutic doses of AAP are considered harmless during these periods, recent findings in both humans and rodents suggest a link between developmental exposure to AAP and behavioral consequences later in life. The aim of this study is to evaluate the impact of neonatal exposure to clinically relevant doses of AAP on adult spontaneous behavior, habituation, memory, learning, and cognitive flexibility later in life using a mouse model. Markers of oxidative stress, axon outgrowth, and glutamatergic transmission were also investigated in the hippocampus during the first 24 h after exposure. In addition, potential long-term effects on synaptic density in the hippocampus have been investigated. In a home cage setting, mice neonatally exposed to AAP (30 + 30 mg/kg, 4 h apart) on postnatal day 10 displayed altered spontaneous behavior and changed habituation patterns later in life compared to controls. These mice also displayed reduced memory, learning and cognitive flexibility compared to control animals in the Morris water maze. An increase of markers for oxidative stress was observed in the hippocampus 6 h after AAP exposure. As AAP is the first choice treatment for pain and/or fever during pregnancy and early life, these results may be of great importance for risk assessment. Here we show that AAP can have persistent negative effects on brain development and suggest that AAP, despite the relatively low doses, is capable to induce acute oxidative stress in the hippocampus.

## 1 Introduction

Paracetamol, or acetaminophen (AAP), is administrated to pregnant woman and neonates as an analgesic and antipyretic. This drug has for a long time been the most common analgesic and antipyretic drug during pregnancy, partly due to its alleged safety. In Northen America and in Northern and Western Europe the prevalence of using AAP during pregnancy is ∼50–60% (Lupattelli et al., 2014). To a large extent, the high AAP use during pregnancy is based on its advantages over other painkillers, with the non-steroidal anti-inflamatoric drugs (NSAIDs) having a less favorable risk-benfit profile in pregnant women (Burdan et al., 2012).

The safety of developmental exposure to AAP has under the recent years been under scrutiny. Epidemiological evidence suggest that developmental exposure to AAP is associated with adverse neurodevelopmental outcomes later in life. More specifically, these studies suggested that there is an association between prenatal AAP exposure and the neurodevelopmental outcomes such as attention deficit hyperactivity disorder (ADHD), autism spectrum disorder (ASD), lower IQ and language delay (Brandlistuen et al., 2013; Liew et al., 2014; Thompson et al., 2014; Avella-Garcia et al., 2016; Liew et al., 2016a; Liew et al., 2016b; Liew et al., 2016c; Stergiakouli et al., 2016; Vlenterie et al., 2016; Bornehag et al., 2018). In animal studies, pre and perinatal AAP exposure have shown to affect neurobehavior later in life. There is a critical period during brain development, the brain growth spurt (BGS), which has been shown to be sensitive to AAP exposure (Viberg et al., 2014; Philippot et al., 2017; Philippot et al., 2018). Among other things, it has been found in mice that therpeutic doses of AAP (30 + 30 mg/kg, 4 h apart, human equvalent dose (HED: 4.9 mg/kg), exposed for a single day during the BGS, change adult spontaneous behavior and habituation rates in a new home environment (Viberg et al., 2014; Philippot et al., 2017). The AAP HED was estimated using the body surface normalization method (Reagan-Shaw et al., 2008). Other studies have shown in rats that continuous fetal and early life exposure to therapeutic doses of AAP (5 or 15 mg/kg, HED: 1.6 or 8.1 mg/kg respectively) change cognitive function, working memory and change neurotransmission in various brain regions (Blecharz-Klin et al., 2015a; Blecharz-Klin et al., 2015b; Blecharz-Klin et al., 2017; Blecharz-Klin et al., 2018).

Regarding the neurodevelopmental adverse effects of AAP*,* the major challenge is its unclear mechanism of action. For its therapeutic use, interaction with the endocannabinoid system (indirect and direct) and the cyclooxygenase system, account for most of AAPs therapeutic effect (Bertolini et al., 2006). However, when it comes to the mechanism that are causing the adverse developmental effects of AAP, at therapeutic doses, many potential mechanisms have been suggested: 1) excess toxic NAPQI formation in the CNS, 2) oxidative stress, inflammation, 3) altered levels of BDNF, 4) effects on endocannabinoid signaling, 5) effects on prostaglandin synthesis, and finally 6) endocrine disruption; for further information read review by Bauer and colleagues (Bauer et al., 2018).

Owing to the uncertain mechanism of action of AAP, and the accumulating indications that exposure to this drug may interfere with brain development, it is important to continue the neurotoxicological evaluation of this commonly used painkiller. In a consensus statement from 2020, a group of researchers (consisting of clinicians, epidemiologists and scientists) state their concern about the recent findings regarding the developmental effects of AAP (Bauer et al., 2021). This statement is based on the accumulating evidence showing increased risks of some neurodevelopmental, reproductive and urogenital disorders if exposed to AAP during pregnancy. Here, the developmental effects on the hippocampus, following PND 10 exposure to AAP, were evaluated by monitoring the time-dependet alterations on oxidative stress markers during the first 24 h after exposure. In addition, protien levels of Glutamate receptor 1 (GLUR1) and Growth Associated Protein 43 (GAP-43) in the hippocamus is measured 24 h after exposure. Then, an evaluation of adult spontaneous behavior, habituation capability, memory, learning and cognitive flexibility was conducted, together with measurments of synaptic marker synaptophysin (SYP) in both the CA3 region of the hippocamus and in the dentate gyrus. By using a singe-day exposure model, information that is relevant for human exposure to AAP is obtained, since exposure to AAP usually are transient.

## 2 Materials and Methods

### 2.1 Animals and Chemicals

Pregnant NMRI mice (from Charles River Laboratory) were purchased from Scanbur, Sollentuna, Sweden and were housed individually in plastic cages in a temperature-controlled (22°C) and light-controlled (12 h light/dark cycle) room with a relative humidity in the range 45–65%. All experimental animals had free access to standardized pellet food (Lactamin, Stockholm, Sweden) and tap water. The pregnant NMRI mice were checked for birth once daily (18.00 h) and day of birth was counted as PND 0. Within 48 h after birth, litter sizes were adjusted to 10–12 pups of both sexes.

AAP (Paracetamol Fresenius Kabi, 10 mg ml^−1^; Fresenius Kabi AB, Sweden; CAS no. 103-90-2) was purchased from Apoteksbolaget, Uppsala, Sweden, and a stock solution containing 6 mg AAP ml^−1^ saline (0.9% sodium chloride in water) was made.

### 2.2 Exposures

Male mice were exposed on PND 10 to either saline vehicle or AAP (30 + 30 mg AAP mg/kg, 4 h apart) with a subcutaneous injection at the scruff.

For the recordings of adult behaviors, male mice at the age of around 4 weeks (after weaning) were separated from their female siblings, which were euthanized, and were kept with their male siblings from each treatment group. Litters contained four to nine animals. When the animals reached 3 months of age they were subjected to spontaneous behavior testing. For biochemical evaluation, male pups, randomly selected from different litters, were administered subcutaneously with either 30 + 30 mg AAP/kg or vehicle on PND 10.

For mechansitical investigations on the neonatal brain, pups were euthanized by decapitation at different time-points during the following 24 h, more specifically following either 2, 6, 12 or 24 h post-dose. Brains were dissected on an ice-cold glass plate and hippocampi were collected and individually snap frozen in liquid nitrogen and then stored at −80°C until assayed.

For mechansitical investigations on the adult brain, adult male mice were anesthetized by intraperitoneal injection of 0.01 mg/g body weight sodium Pentobarbital (Apoteket Farmaci, Sweden) and the tissues were fixed by transcardiac perfusion with 4% formaldehyde (Histolab, Sweden). The brains were then dissected out and were stored in 4% formaldehyde overnight before they were frozen in CryMold and stored at −80°C until sectioning.

### 2.3 Behavior Assessment

#### 2.3.1 Spontaneous Behavior and Habituation Rates in a New Home Cage in 3-month Old Mnice

Mice exposed to AAP (30 + 30 mg/kg, 4 h apart; *n* = 9) and control mice (vehicle; *n* = 9) were tested for spontaneous locomotor activity in a new home cage. For this, the Panlab Infrared (IR) Actimeter was used. The system is composed by a 2-dimensional (*X* and *Y*-axes) square frame. Each frame counts with 16 × 16 infrared beams for subject detection, where activity from four cages were analyzed simultaneously. Spontaneous behavior in a novel home environment measures the integration of sensory input into motor output and tests the animals’ ability to integrate new information with information previously attained, hence the mice ability to habituate. Habituation is a non-associative form of learning and is considered cognitive function (Groves and Thompson, 1970; Daenen et al., 2001; Wright et al., 2004). The ability to habituate to a new environment is here defined as a decrease in distance travelled (mm) over time. The concept of using a decrease in activity, when introduced to a new home cage, has been explained in more details elsewhere (Fredriksson, 1994; Eriksson et al., 2016). In breif, the test was conducted when mice reached 3 months of age and observations took place between 08:00 and 12:00 h, under the same light and temperature conditions in which the animals were housed. Four individuals were randomly chosen from three to four different litters from each exposure group. Control mice reached base-line activity after 90 min of habituation. In each 90 min observation session, recordings of the distance travelled were then sub-devided into three equally long time spells, and subsequently statistically evaluated.

#### 2.3.2 Memory, Learning and Re-learing Abilities Using the Morris Water Maze in 4-month Old Mice

Mice exposed to AAP (30 + 30 mg/kg, 4 h apart; *n* = 12) and control mice (vehicle; *n* = 10) were tested in a swim maze at 4 month of age. These animals were randomly chosen from three to four different litters. The animals were tested in a swim maze of the Morris water maze (MWM) type (Morris et al., 1982), as previously described (Viberg et al., 2003).

In breif, a circular container with a diameter of 103 cm was filled with water to a depth of 15 cm from the brim (water temperature 21°C). External visual cues were positioned on the north, south, east and west walls; the relative positions of the observer and the Morris maze pool were the same throughout the course of the swim maze test. In the middle of the northwest quadrant a metal mesh platform with a diameter of 12 cm was submerged 1 cm below the water surface. The behavioural test was performed on five consecutive days to test the mouse’s spatial learning ability to locate the platform on the first 4 days (five trials/day). On the fifth day, the platform was re-located to the northeast quadrant and the mice were tested for their re-learning ability (five trials), otherwise the procedure was identical. During the first 4 days of aquisituion, the latancy to find the platform was used to assess spatial learning performance. Following re-location of the platform, the latency to find the platform’s new position was used to assess cognitive flexibility.

Each mouse was placed on the platform for 20 s and then released, head toward the wall of the container, in different squadrants on a rotating schedule (southwest → southeast → northeast → southwest), starting as follows: southwest quadrant on day 1; southeast squadrant on day 2; northeast squadrant on day 3; southwest sqadrant on day 4. The mice had 30 s to locate the submerged platform, and between each trial the mouse rested on the platform for 20 s. The time taken to reach the platform was measured by the observer.

### 2.4 Biochemical and Molecular Assessment

#### 2.4.1 Hippocampal Oxidative Stress Using Quantitative Real-Time PCR 2, 6, 12 and 25 h After Exposure

Relative expression levels of mRNAs were measured by quantitative real-time PCR. Hippocampus was homogenized in PureZOL isolation agent (BioRad, Stockholm, Sweden). The total RNA was isolated using Aurum Total RNA extraction columns (Bio-Rad, Stockholm, Sweden) and treated with DNAse to remove possible genomic DNA contamination, according to the manufacturer’s instructions and stored at −80°C. Reverse transcription to cDNA was done using iSCRIPT (BioRad, Stockholm, Sweden). Gene transcription of *Nuclear factor (erythroid-derived 2)-like 2* (*Nrf2*; encoded by *Nfe2l2* gene) and *Kelch-like ECH-associated protein 1* (*Keap1*) was normalized against transcription of housekeeping genes *Phosphoglycerate kinase 1* (*Pkg-1*) and *Glyceraldehyde 3-phosphate dehydrogenase* (*Gapdh*)*.* All primer sequences area shown in Table 1. The efficiency of each primer pair was determined from a standard curve with pooled cDNA. Annealing temperature was 62°C. Gene transcription analyses of the genes of interest were analyzed with the 2^−ΔCt^ method. Each sample was run as a duplicate. To ensure the amplification of a single product, a melt curve for each qPCR reaction was performed (melt curve ranging from 55°C to 95°C).

**TABLE 1:** Gene-Specific Primer Sequences Used for qPCR.

Target name	Accession No.	Forward primer (5′–3′)	Reverse primer (5′–3′)
Gapdh	NM_008084.3	GGG​CTC​CCT​AGG​CCC​CTC​CTC​TTA​T	CAC​CCC​AGC​AAG​GAC​ACT​GAG​CAA​G
*Pgk-1*	NM_008828.3	CTC​CGC​TTT​CAT​GTA​GAG​GAA​G	GAC​ATC​TCC​TAG​TTT​GGA​CAG​TG
*Nrf2*	NM_010902.4	GCC​CAC​ATT​CCC​AAA​CAA​GAT	CCA​GAG​AGC​TAT​TGA​GGG​ACT​G
*Keap1*	NM_016679.4	TGC​CCC​TGT​GGT​CAA​AGT​G	GGT​TCG​GTT​ACC​GTC​CTG​C

#### 2.4.2 Hippocampal Levels of GAP-43 and GLUR1 Using Western Blot 24 h After Exposure

A phosphatase and protease inhibitor tablet (A32959, ThermoScintific) was dissolved into 10 ml ice cold 1xPBS solution and it was used with a ratio of 1 ml/100 mg tissue for each sample. Glass beads were then added to the samples with a ratio of 1:1. A bullet blender was then used for sample homogenization. In order to eliminate the cell membranes, −80°C freeze and thaw cycles were executed twice. The homogenate was centrifuged for a duration of 5 min at 5,000 rpm at 4 c and stored at −20°C.

The protein concentration in the samples were then determined with BCA technique. Standard solutions (BSA) and a working reagent (BCA) were prepared. Duplicates of 10 ul/sample and 10 ul/standard solution were added in wells in a microplate. Afterwards, the working reagent was added to each well. The sample incubated at 37°C for 20 min. The absorbance was measured at 562 nm.

Western blot was used to analyze the proteins of interest: GAP43 and GLUR1. The western blot procedure was done two times for each marker, the first time with three different protein concentration for optimization, and then a second time was performed on the most optimal protein concentration. For sample protein separation, the supernatants were used in three different concentration (undiluted, 1:5 diluted and 1:10 diluted) for optimization or the most optimal concentration. A sample buffer was prepared, containing beta-mercaptoethanol (Sigma-Aldrich) and laemmli sample buffer (Bio-Rad). This was mixed with the supernatants with 1:1 ratio and incubated at 97°C for 8 min. The samples were then cooled at room-temperature. These and a prestained protein ladder (ThermoScintific) were loaded on the wells of a Mini-PROTEAN (R) TGX Stain-Free ™ 6–16 gel (456–8,106, Bio-Rad). Then the electrophoresis was running at 120 V for 1 h. For total protein measurment, the gel was removed and imaged using a Gel DocTM imager (Bio-Rad). The proteins in the gel were transferred to a Trans-Blot TurboTM Mini PVDF membrane (1,704,156 BioRad) using a Trans Blot ® TurboTM (BioRad). Then the membrane was incubated on strong agitation at RT for 1 h in a blocking buffer consist of Tween^Ⓡ^ 20 (Sigma-Aldrich) 1xTBS buffer pH 8 (0.1 M Trizma^Ⓡ^base (Sigma-Aldrich) 1.5 M NaCl (Sigma-Aldrich) and dH2O) and 5% Blotting Grade Blocker nonfat dry milk (BioRad). Primary antibody for the protein of interest was diluted in the blocking buffer and used for membrane overnight incubation at 4°C with gentle agitation. The experiment was continued the next day with membrane rinsing followed by a washing step in 1xTTBS 3 × 10 min with strong agitation. Afterwards a HRP-conjugated secondary antibody was diluted 1:10,000 with the blocking buffer and used for 1 h membrane incubation with gentle agitation at RT. A 1:1 mixture of Luminol/enhancer and peroxidase buffer solution (Immuno star HRP BioRad) was prepared and used for membrane incubation at RT for 3 min without agitation. Membrane imaging was performed using a CCD camera (Chemidoc MP BioRad and image Lab 5.5 system) for chemiluminescence.

#### 2.4.3 Hippocampal Synaptic Density Using Immunohistochemisry in 4-month-Old Mice

In this experiment, the frozen brains were cut in coronal sections (10 μm) using a CryoStar™ NX70 Cryostat (Thermo Scientific™) at −20 ± 1°C, thaw-mounted on SuperFrost® Plus slides (Menzel-Gläser, Germany) and stored at −20°C until examination by an immunohistochemical method.

Sections from the hippocampus region (bregma −1.94) were boiled in 0.01 M citric acid for 5 min. After boiling, the sections were marked by “Pap pen” and then washed 3 × 5 min in phosphate-buffered saline (PBS). The sections were then covered by primary antibodies diluted in Supermix (200 ml TBS, 0.5 g gelatin, 1 ml Triton X-100) and placed in a humidity chamber at 4°C overnight. The optimal concentration for synapotophysin (SYP) primary antibody was identified (1:100) through optimization. The next day the sections were washed 3 × 10 min in PBS and were incubated for 2 hours at room temperature in a humidity chamber in the dark with secondary antibody diluted in Supermix. The secondary antibodies used were anti-mouse diluted in 1:400 Supermix. The sections were then washed 3 × 5 min in PBS and each section was then mounted with 50 μL of ProLong Gold antifade reagent with DAPI and kept in the fridge until microscope analysis. The quality of the sections and autofluorescence was controlled to make sure and identify that it was the antibody that gave the signal.

Images for SYP were taken using a Zeiss AxioImager M2 (Zeiss, Germany) at the BioVis platform, Uppsala University. Fluorescent signals were collected at 488 nM for SYP with an fixed exposure time of 2.62 s. Immunofluorescence intensity per area unit (A.U.) was blindly quantified using ImageJ. For each individual five to six measurements were used to establish a mean value. The mean value for each individual was then considered the statistical unit.

### 2.5 Statistical Analysis

Normality of residuals and homogeneity of variances were checked using QQ-plots and homoscedasticity-plots, respectively; if needed, data were log-transformed to meet the assumptions of parametric statistics. In the analysis of behavior, a 2-way RM ANOVA was used to investigate if there was 1) an effect of the within-subject factor (i.e., time), 2) an effect of the between-subject factor (i.e., treatment), and 3) if there was interaction-effect between the within-subjects factor and between-subjects factor (i.e., treatment × time) on the dependent variable. In the analysis of MWM data, the total time over the five consecutive trails/day was compared between conditions. Following ANOVAs, Šidák’s multiple comparisons test was used. When two means were compared, a student’s t-test was used. Effect sizes for ANOVAs and t-tests were calculated for using partial eta-squared (η_*p*_^*2*^ *;* https://effect-size-calculator.herokuapp.com/#form4) and Cohen’s d (*d;* https://www.socscistatistics.com/effectsize/default3.aspx), respectively. Graphical illustrations, normality testing and analyses of equal variances were made in GraphPad Prism version 8.0.2 and version 5.01 (GraphPad software Inc., CA, United States).

## 3 Results

There were no signs of toxic symptoms in any of the mice during the experiments. Body weights were measured on PND 10 and at sacrifice (for both mice euthanized within the first 24 h after exposures and mice raised until adulthood). There were no significant effects of exposures on body weights in either neonates (*p* > 0.05) nor in mice raised to adulthood (*p* > 0.05).

### 3.1 Spontaneous Behavior in a New Home Cage

**FIGURE 1:**
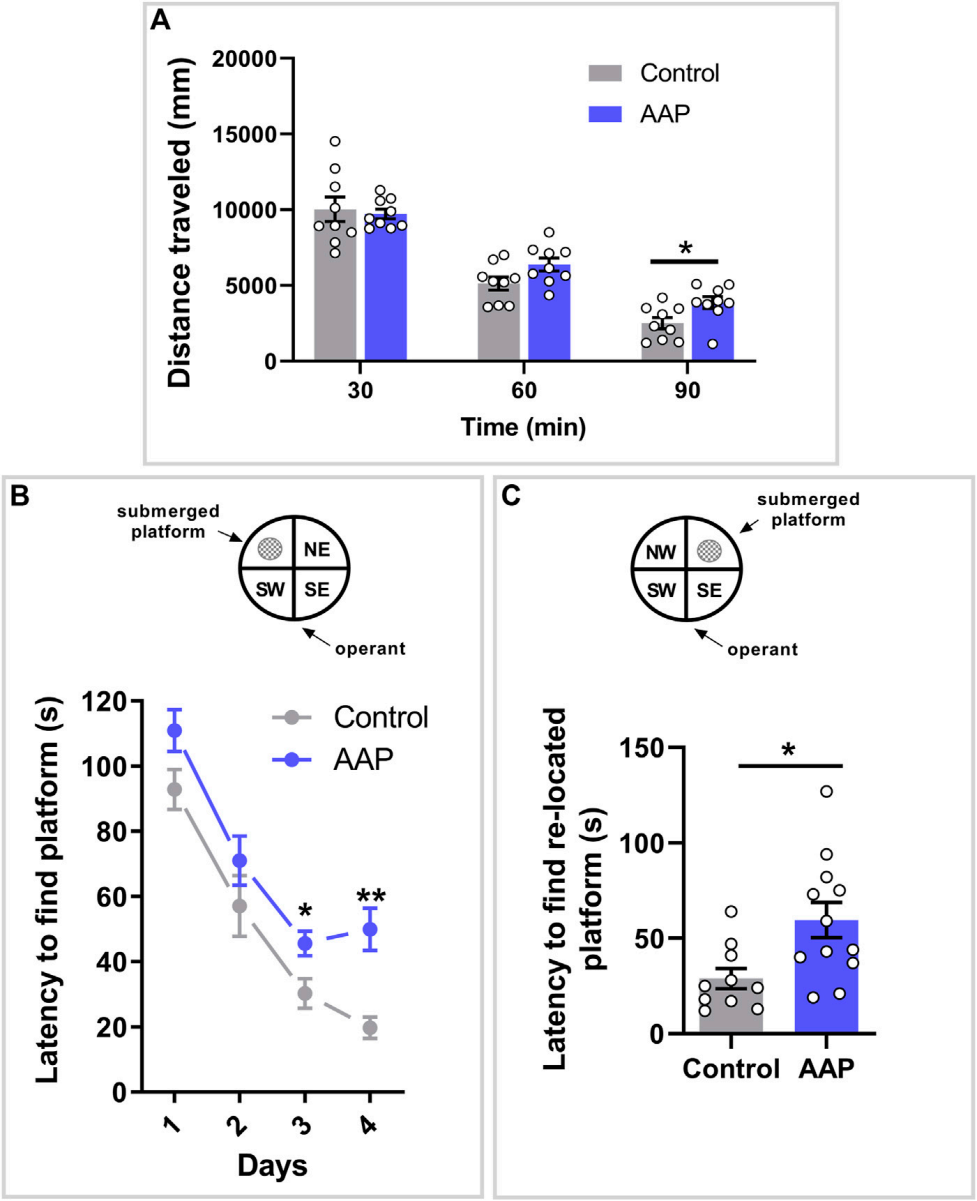
Behavioral assessments in adulthood following neonatal exposure to AAP. **(A)** Spontaneous behavior in 3-month-old mice exposed to vehicle or AAP (30 + 30 mg/kg, 4 h apart) on PND 10. Distance traveled (mm) during each of the three time spells were measured when the mice were introduced to a new home cage (*n* = 10). **(B)** Swim maze performance in four-month-old mice (*n* = 10–12) exposed to vehicle or AAP (30 + 30 mg/kg, 4 h apart) on PND 10. Latencies (s) to locate the platform were measured during the 4 days acquisition period (trials 1–20) and **(C)** during re-learning period on the fifth day (trials 21–25). Statistical differences are indicated as * if significantly different vs. controls *p* ≤ 0.05 and ** if significantly different vs. controls *p* ≤ 0.01. Bars represent means ± SEM.

A significant interaction between treatment and time was shown on the dependent variable distance, F (2, 32) = 3.575, *p* = 0.04, η_*p*_^*2*^ = 0.18 (Figure 1A). Post-hoc analyses using Šidák’s multiple comparisons test revealed a significant difference between control mice and AAP exposed mice during the last 30 min of behavior recordings (*p* = 0.01) (Figure 1A).

### 3.2 Memory, Learning and Cognitive Flexibility in the Morris Water Maze

A significant interaction between treatment and time was indicated on the latency to find the submerged platform, F (3, 60) = 3.552, *p* = 0.020, η_*p*_^*2*^ = 0.15. Post-hoc analyses using Šidák’s multiple comparisons test revealed a significant difference between control mice and AAP exposed mice on both day 3 (*p* = 0.037) and 4 (*p* < 0.0001) (Figure 1B). An independent t-test indicated that mice neonatally exposed to AAP used significantly more time to find the re-located platform compared to control mice on day 5, *t* (20) = 2.96, *p* = 0.0078, *d* = 1.6 (Figure 1C).

### 3.3 Hippocampal Oxidative Stress

**FIGURE 2:**
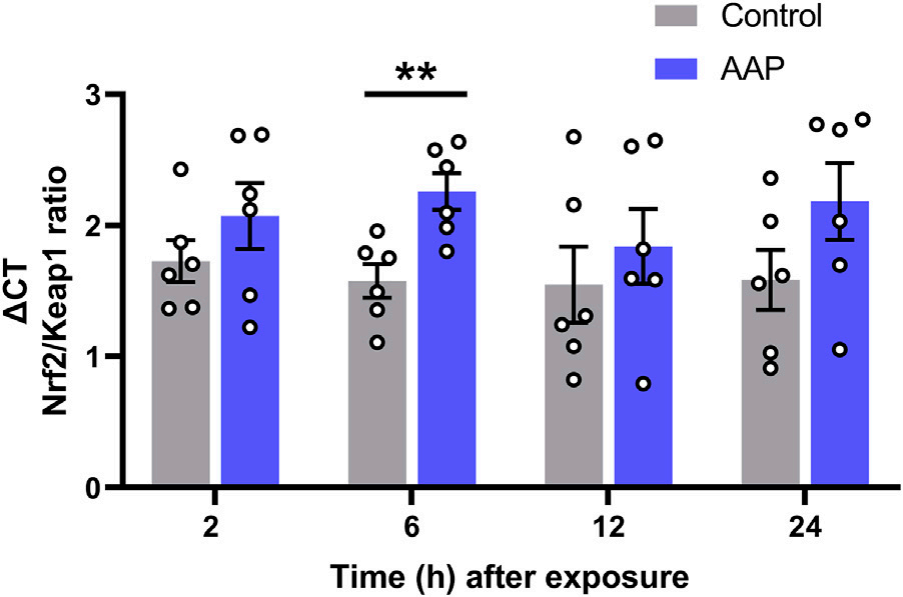
Transcript level ratios of the genes *Nrf2* and *Keap1* 2, 6, 12, and 24 h after exposure to vehicle or AAP (30 + 30 mg/kg, 4 h apart) on PND 10. ΔCT in *Nrf2/Keap1* expression ratio is relative to that of the corresponding untreated mice at each time point (not shown). Statistics were done on the raw data (ΔCT) at each time point. **, *p* < 0.005 (by Student’s t test). Bars represent means ± SEM of six mice.

A significant increase in the *Nrf2/Keap1* transcriptional ratio was shown 6 h after exposure to AAP (30 + 30 mg/kg, 4 h apart), *t* (10) = 3, *p* = 0,0050, *d* = 0.21, in the hippocampus (Figure 2). No effects were observed on *Nrf2/Keap1* transcriptional ratio 2, 12 or 24 h after exposure.

### 3.4 Hippocampal Levels of GAP-43 and GLUR1

**FIGURE 3:**
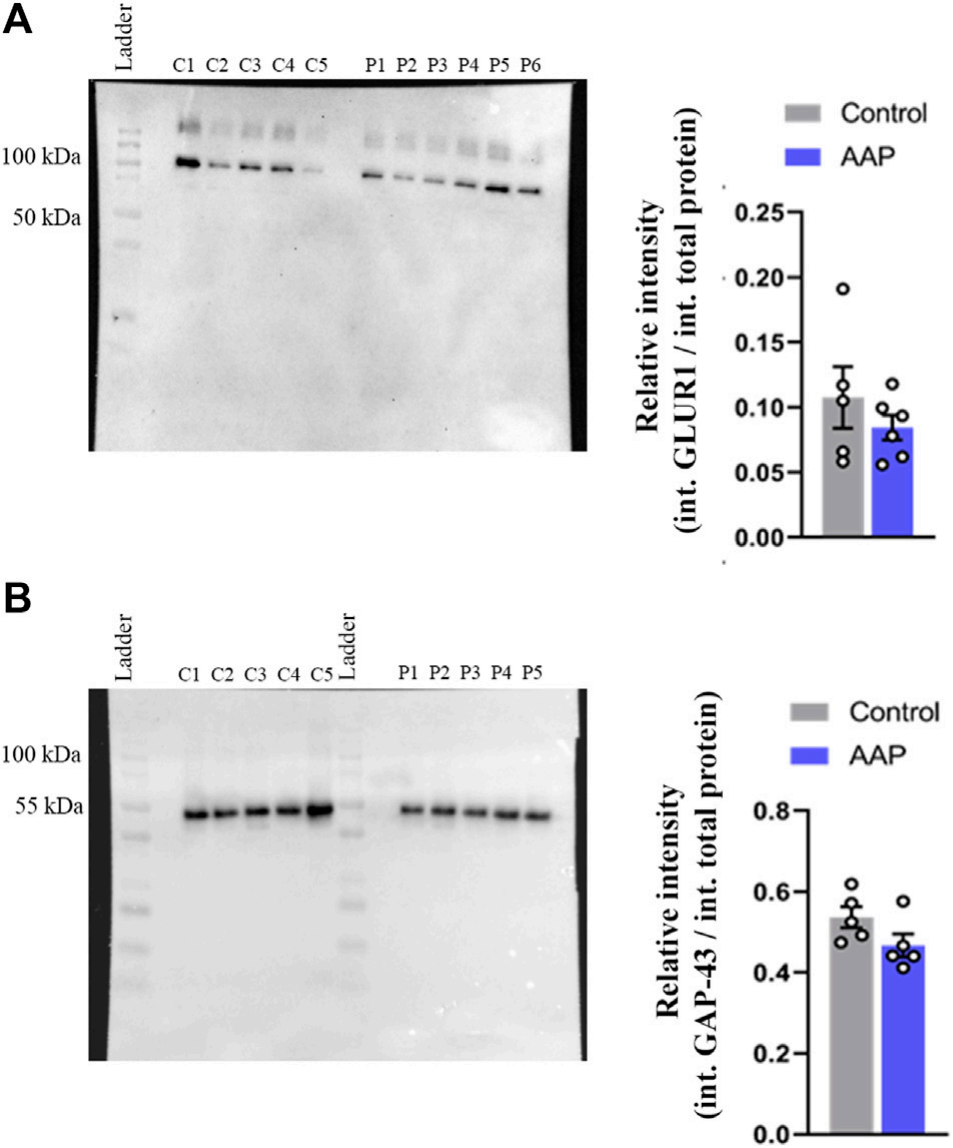
Protein levels, presented as relative intensity of total protien levels, of **(A)** GLUR1 and **(B)** GAP-43 of male NMRI mice 24 h after exposure to either vehicle (control) or 30 + 30 mg/kg AAP. “C1-5” represent control samples in the blot; “P1-6” represents AAP samples in the blot. The height of the bars represent the mean ± SEM value of five to six animals.

No effects were observed on neither GAP-43, *t* (8) = 1.78, *p* = 0.11, nor GLUR1, *t* (9) = 0.97, *p* = 0.36, levels in the hippocampus using Western blot (Figure 3). However, a large effect size was observed for GAP-43, *d* = 1.13, and a medium effect size was observed for GLUR1, *d* = 0.6.

### 3.5 Hippocampal Synaptic Density

**FIGURE 4:**
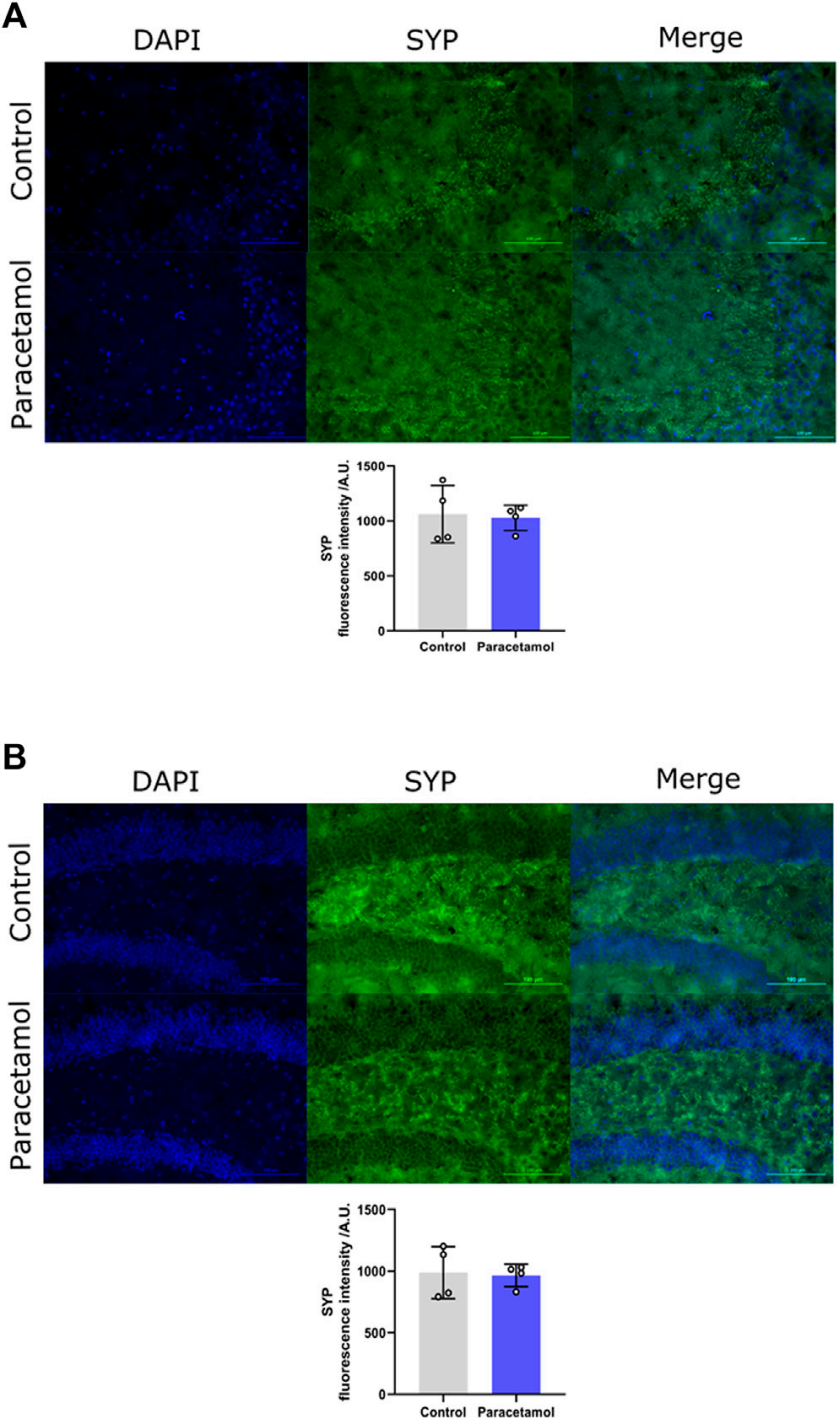
Immunofluorescence staining of SYP in **(A)** CA3 and **(B)** DG coronal sections of the adult mouse hippocampus (Bregma −1.94). Nucleic staining with DAPI accompanied by immunological staining for SYP. Scale bar = 100 µm. Histogram show relative immunofluorescence intensity/A.U. of SYP measured in male adult mice after neonatal exposure to either saline vehicle or AAP on PND 10 in CA3 and DG. *p* > 0.05. The height of the bars represent mean value ±SEM. Each exposure group contained *n* = 4 mice.

Immunohistochemical evaluation did not reveal any differences in synaptophysin levels of the CA3 region, *t* (6) = 0.24, *p* = 0.8217, *d* = 0.17, nor in the dentate gyrus region *t* (6) = 0.20, *p* = 0.85, *d* = 0.14, between AAP exposed animals and controls (Figures 4A,B, respectively).

## 4 Discussion

This study reports of developmentally induced neurotoxic effects following exposure to relevant doses of AAP in mice. Mice neonatally exposed to AAP displayed impaired habituation capability, reduced memory and learning ability, and decreased cognitive flexibility in adulthood. These behavioral effects were observed in the adult mice following a single day exposure. These effects could potentially depend on increased hippocampal oxidative stress, as indicated by the observed increase in *Nrf2/Keap1* transcriptional ratio 6 h after exposure.

We administered AAP (30 + 30 mg/kg, 4 h apart) into ten-day-old mice pups. This dose, which is corresponding to a clinically relevant dose in humans (HED: 4.9 mg/kg), has previously been demonstrated to lead to adverse behavioral effects in adulthood (Viberg et al., 2014; Philippot et al., 2017; Philippot et al., 2018). The recommended dose of AAP in newborns and toddlers is 7.5–15 mg kg^−1^ up to four times a day (Anderson and Allegaert, 2009). Ten-day-old mice, in terms of key developmental processes, are comparable to the time around birth in humans (Dobbing and Sands, 1979; Semple et al., 2013).

The spontaneous behavior test, when mice are introduced to a new home cage, explores the animal’s ability to habituate to a new environment. The mice have to integrate a sensory input into motor output, but as the stimulus (e.g., the new environment) becomes more familiar, the response (explorative activity; here as distance travelled) gradually decreases. Control animals reached base-line activity following 90 min in the new home cage. Mice that neonatally were exposed to AAP displayed an increased activity during the last 30 min of recording compared to these controls. Habituation is considered a non-associative form of learning, hence is considered a cognitive function (Groves and Thompson, 1970; Daenen et al., 2001; Wright et al., 2004). Previous findings following developmental exposure to AAP in rodents have also indicated both a hypo-responsiveness to a new environment and effects on memory and learning (Blecharz-Klin et al., 2017; Philippot et al., 2017; Philippot et al., 2018). In line with this observation, it has been shown that ASD patients display hypo-responsiveness to novel stimuli (van Engeland et al., 1991; Webb et al., 2010). The timing of AAP exposure during the BGS also seems to be of essence for the neurotoxicity in mice. In a study from 2017 (Philippot et al., 2017), AAP exposure during neurodevelopmental time points corresponding to late pregnancy/early life and the beginning of the third trimester in humans altered adult habituation and spontaneous behavior in mice. On the other hand, in the same study exposure during a time point corresponding to a two-year-old child did not affect adult behaviors in mice. It has already been pointed out that AAP exposure late in pregnancy seem to be closely related to adverse outcomes later in life in humans (Bauer et al., 2018).

To further investigate effects on memory, learning and cognition, the MWM was used. During the 4-day acquisition period, control animals reduced the time needed to locate the submerged platform. This test showed that mice exposed to AAP on PND 10 displayed a significant increase in latency to reach the platform compared to control animals during day 3 and 4 of the acquisition phase. As this indicates an impact on memory and learning, it also confirms previous findings that have shown that AAP exposure during brain development affects cognition (Viberg et al., 2014; Blecharz-Klin et al., 2017; Blecharz-Klin et al., 2018). To assess cognitive flexibility in AAP-treated mice, a cognitive process that to our knowledge has not yet been explored in experimental animals following AAP administration, the platform in the MWM was re-located to a new quadrant. The latency to find the new position was measured over five trials (on day 5). Mice that neonatally received AAP displayed an increased latency to find the re-located platform compared to controls, thus indicating decreased ability re-learn a new task previously learned. It therefore seem that neonatal exposure to AAP affects both learning in a constant environment and adapting flexibly to a changing one.

As there are several suggestions to possible mechanisms of action in the developmental effects observed following exposure to AAP (Bauer et al., 2018), more studies to investigate each and one of these suggestions are needed. Furthermore, there is no reason to see all of these hypothetical developmental neurotoxic mechanisms as very independent mechanisms, as many of these may depend on one another. AAP interacts with the endocannabinoid system through one of its active metabolites (Högestätt et al., 2005). It has been shown that AAPs interaction with the CB1R is essential in its analgesic properties (Ottani et al., 2006). There is also a known interaction between endocannabinoid system and the neurotrophic factor BDNF and its receptor TRKB (Berghuis et al., 2005; D’Souza et al., 2009). As our research group have observed altered transcript levels of *Trkb* following PND 10 exposure to both AAP (Philippot et al., 2018) and THC (Philippot et al., 2019), together with the facts that both these drugs/pharmaceuticals interact with the endocannabinoid system, we emphasize the need to thorough investigate the endocannabinoid-mediated effects of AAP on brain development. This endocannabinoid system is known to be involved during brain development and expressed in most part of the brain (Fernández-Ruiz et al., 2004; Berghuis et al., 2007; Harkany et al., 2007; Mulder et al., 2008). Interestingly, concomitant activation of the endocannabinoid system has been shown to increase sensitivity to AAP during brain development in mice (Philippot et al., 2018). On a neuronal level, the endocannabinoid receptors are expressed, and act in a modulatory fashion, in most signaling system: serotonergic, dopaminergic, cholinergic (Hermann et al., 2002; Häring et al., 2007; Oropeza et al., 2007; Azad et al., 2008; Kano et al., 2009; Morozov et al., 2009). The endocannabinoid system have been shown to be involved in the development of ASD (Földy et al., 2013; Brigida et al., 2017; Zamberletti et al., 2017). There is also an association between a polymorphism in the endocannabinoid-degrading enzyme FAAH with ADHD (Ahmadalipour et al., 2020). By affecting the endocannabinoid system there is a risk that other transmitter systems also may be affected and be one possible reason for the variety of observed effect on neurodevelopment already observed following AAP (Blecharz-Klin et al., 2015a; Blecharz-Klin et al., 2015b; Blecharz-Klin et al., 2017; Blecharz-Klin et al., 2018). In line with our proposed hypothesis of a CB1R/BDNF/TRKB mediated developmental neurotoxicity, BNDF have been associated with ADHD in humans (Dark et al., 2018). Effects on BDNF have also been found after developmental exposure to AAP in rodents (Viberg et al., 2014; Blecharz-Klin et al., 2018). In humans, dysregulation of BDNF has been found to be involved in ASD and ADHD (Bryn et al., 2015; Liu et al., 2015). Furthermore, single-nucleotide variants of BDNF have been found to be associated with increased risk of ADHD (Kent et al., 2005; Hawi et al., 2016). AAP also affects the cyclooxygenase (COX) system. However, an interaction as such seem less likely in developmental neurotoxicity observed following PND 10 exposure to AAP since PND 10 exposure to ibuprofen (with a known interaction with the COX system) did not alter adult spontaneous behavior and habituation capabilities (Philippot et al., 2016).

The antioxidant response element (ARE) are involved in trascription of numerous genes involved in detoxification and cytoprotection (Kensler et al., 2007). The trascription factor NRF2 drives the ARE-mediated trascription of cytoprotective elements (Itoh et al., 1997). Under homeostasis, cytocolic NRF2 is inactivaded by forming a complex with KEAP1 (Itoh et al., 2003); however, during oxidative stess the aforementioned complex is disrupted and NRF2 is translocated into the nucleus to bind ARE, in turn leading to expression of serveral cytoprotective proteins. An increase in cytoprotective elements, as indicated by an increase in the *Nrf2/Keap1* trascriptional ratio, was showed following PND 10 AAP exposure in the hippocampus 6 h after exposure. This suggests that relevant doses of AAP is causing oxidative damage to the developing mouse brain, which, in part, could be one of the underlying reasons for the behavioral manifestations observed herein. Interetingly, an increase of these cytoprotective elements has already been observed following PND 10 exposure to THC (50 mg/kg) 24 h after exposure (Philippot et al., 2019). In line with these observations, high doses of AAP has been shown to increase ROS formation leading to mitochondrial dysfunction (da Silva et al., 2012). High doses of AAP seem to induce transcript levels of oxidative stress response genes in mice brains (Ghanem et al., 2015). Oxidative stress and inflammatory responses have been implicated in the etiology of ADHD and ASD, as indicated by differential expression of pro-inflammatory cytokines (Goines et al., 2011; Jones et al., 2017). Interestingly, therapeutic doses of AAP seemed to activate oxidative stress-related gene responses, similar to those observed following higher doses, in humans (Jetten et al., 2012).

Synaptic organisation is crusial during brain development. For example, GAP-43 is vital for axonal growth and is constantly used as a biomarker for axonal outgrowth. The expression of GAP-43 is reaching a peak during brain development, indicating its fundamental role during brain development (Oestreicher et al., 1997). Thus, effects on axonal outgrowth during brain development may result in different synapse density later in life. For this reason, the neonatal mice were examined for GAP-43 24 h after AAP exposure and adult animals were examined for the presynaptic marker SYP in both CA3 and dentate gyrus. We show here that neither neonatal levels of GAP-43 nor SYP levels in the adult animal have been affected after early AAP exposure. An investigation of hippocampal GLUR1 was also conducted 24 h after exposure. GLUR1 is known as the most expressed subunit of alfa-amino-3-hydroxy-5-methyl-4 isoxazolepropionic acid (AMPA) receptor (Wenthold et al., 1996). AMPA receptor is essential for synaptic plasticity (Lee et al., 1998). Since synaptic plasticity of excitatory synapse is considered to be crucial for the brain, in particular, for memory and learning processes, possible effects on GLUR1 followin neonatal AAP exposure could give insights into AAPs effect on memory and learning. However, no effect on GLUR1 protein levels was observed herein.

As previously mentioned, other experimental studies that investigated the effect of AAP on rodent brain development have shown the effect on many neurotransmitter systems (Blecharz-Klin et al., 2015a; Blecharz-Klin et al., 2015b; Blecharz-Klin et al., 2017; Blecharz-Klin et al., 2018). However, the clinical relevance has been questioned as the exposure occurred continuously for several weeks and therefore would not be clinically representative to human exposures (Mian and Allegaert, 2017); the brain development of rats and mice is more compressed compared to humans, thus weeks of exposure in rodents will correspond to unrealistic exposures in humans. Our exposure model therefore provides toxicological data relevant to humans and that are highly warranted, as AAP remain the first line treatment for pain and fever during pregnancy and early life.

The long term neurodevelopmental effects of AAP have been studied in an increasing amount of epidemiological studies, where AAP exposure during pregnancy have shown associations with ADHD, ASD, lower IQ and language delay (Bauer et al., 2018). These associations have been found in different populations: the Norwegian Mother and Child Cohort Study (MoBA) (Brandlistuen et al., 2013; Vlenterie et al., 2016), the Danish National Birth Cohort (DNBC) (Liew et al., 2014; Liew et al., 2016a; Liew et al., 2016b; Liew et al., 2016c), the Auckland Birthweight Collaborative Cohort (Thompson et al., 2014), the Spanish Infancia y Medio Ambiente (INMA project) (Avella-Garcia et al., 2016), the Avon Longitudinal Study of Parents and Children (ALSPAC) (Stergiakouli et al., 2016) and the Swedish Environmental Longitudinal, Mother and child, Asthma and allergy (SELMA) (Bornehag et al., 2018). The fact that these associations have emerged from a whole range of different populations strengthens the connection between AAP exposure and its negative effects on brain development. There is however a need to confirm these associations with mechanistic data. In the consensus statement by Bauer and colleagues (Bauer et al., 2021), authorities such as the EMA and the FDA are urged to review all available literature, both epidemiological and non-clinical. The results presented in this article can contribute to a further understanding of AAP’s developmental neurotoxic potential and will hopefully be able to be part of making a more accurate risk/benefit assessment of its usage during pregnancy. In summary, this study have shown that clinically relevant doses of AAP affect adult memory, learning and cognitive flexibility in mice, possibly by inducing oxidative damege in the hippocampus. These results are important as AAP is the only recomennded painkiller during pregnancy and that the adverse effects were induced following only a single day exposure, thereby may be representative to a human exposure setting. We also suggest that AAP, despite the relatively low doses, appears to induce oxidative stress in the hippocampus. However, furhter insight in the mechansim resposible for these effects are highly warranted.

## Data Availability

The raw data supporting the conclusion of this article will be made available by the authors, without undue reservation.
